# A systematic review and meta-analysis to assess the association between urogenital schistosomiasis and HIV/AIDS infection

**DOI:** 10.1371/journal.pntd.0008383

**Published:** 2020-06-15

**Authors:** Ludoviko Zirimenya, Fatima Mahmud-Ajeigbe, Ruth McQuillan, You Li

**Affiliations:** 1 Medical Research Council/Uganda Virus Research Institute & London School of Hygiene and Tropical Medicine Uganda Research Unit, Entebbe, Uganda; 2 University of Edinburgh, Scotland, United Kingdom; 3 Ahmadu Bello University Teaching Hospital, Shika-Zaria, Nigeria; London School of Hygiene and Tropical Medicine, UNITED KINGDOM

## Abstract

**Background:**

Urogenital schistosomiasis and HIV/AIDS infections are widespread in sub-Saharan Africa (SSA) leading to substantial morbidity and mortality. The co-occurrence of both diseases has led to the possible hypothesis that urogenital schistosomiasis leads to increased risk of acquiring HIV infection. However, the available evidence concerning this association is inconsistent. The aim of this study was to systematically review and quantitatively synthesize studies that investigated the association between urogenital schistosomiasis and HIV/AIDS infection.

**Methods:**

A systematic review basing on PRISMA guidelines was conducted. It is registered with PROSPERO, number CRD42018116648. We searched four databases, MEDLINE, EMBASE, Global Health and Global Index Medicus for studies investigating the association between urogenital schistosomiasis and HIV infection. Only studies published in English were considered. Results of the association were summarised by gender. A meta-analysis was performed for studies on females using random-effects model and a pooled OR with 95% confidence interval was reported.

**Results:**

Of the 993 studies screened, only eight observational studies met the inclusion criteria. Across all studies, the reported unadjusted OR ranged from 0.78 to 3.76. The pooled estimate of unadjusted OR among females was 1.31 (95% CI: 0.87–1.99). Only four of the eight studies reported an adjusted OR. A separate meta-analysis done in the three studies among females that reported an adjusted OR showed that the pooled estimate was 1.85 (95% CI: 1.17–2.92). There were insufficient data to pool results for association between urogenital schistosomiasis and HIV infection in the males.

**Conclusion:**

Our investigation supports the hypothesis of an association between urogenital schistosomiasis with HIV/AIDS infection in females. Due to insufficient evidence, no conclusion could be drawn in males with urogenital schistosomiasis. Large-scale prospective studies are needed in future.

## Author summary

Urogenital schistosomiasis, caused by parasitic trematode *Schistosoma haematobium* is a significant source of morbidity in sub Saharan Africa. HIV infection caused by a retrovirus is of two subtypes HIV 1 and HIV 2, with subtype HIV 1 being found worldwide and more aggressive, leading to HIV/AIDS. Research on both of these diseases in the same settings, has shown that these diseases cross paths. This has led to the suggestion that there could be a possible association between the two. Here we describe a systematic review that was carried out to determine if there is an association between UGS and HIV/AIDS infections. We searched all published articles available in MEDLINE, EMBASE, Global Health (CABI), and Global Index Medicus before 28^th^ January 2020. We found eight observational studies eligible to be included in the systematic review and no intervention study. Six of these studies were included in the meta-analysis. A summarized meta-analysis of the study findings with adjusted OR showed that there was a likely association between urogenital schistosomiasis and HIV/AIDS infections in females. However, due to limited papers in males, no conclusion could be drawn.

## Introduction

Schistosomiasis is a disease of public health importance and causes a high burden of morbidity, with an estimated 3.06 million disability adjusted life years annually [1]. WHO estimates that the highest proportion of schistosomiasis in Sub Sahara Africa (SSA) is due to *S*. *haematobium* with 112 million infected and 436 million at risk of infection [2]. As *S*. *haematobium* infection causes both urinary and genital pathologies, in 2009, WHO recommended that this infection should henceforth be referred to as urogenital schistosomiasis (UGS) [3]. About 75% of women in SSA with *S*. *haematobium* infections have vulva, vagina and cervical ulceration secondary to urogenital schistosomiasis [4]. In males, ulcerative lesions have been observed in the seminal vesicle lumen with mucosal thickening and enlargement [5].

The spread of both HIV/AIDS and UGS has been shown to cross paths in SSA populations [6]. This has led to the hypothesis of a possible association between the two diseases, and particularly, whether UGS could predispose individuals to HIV/AIDS [7]. This association has been thought to occur through different mechanisms such as breakage of urogenital epithelial lining, triggering of pro inflammatory changes and increase of HIV target cells that are usually coupled with increased genital vascularity[8–13]. Schistosomiasis has further been found to elicit an immune response that affect ways in which the body responds to other infections. Immune response following schistosomiasis is dominantly a Th2 response, this has been shown to impair Th1 response that is key in fighting off bystander antigens like the HIV virus [14].

Mathematical modelling has further demonstrated that schistosomiasis may increase the prevalence of HIV/AIDS in settings where both infections co-exist [15]. In a study that compiled country level data for 43 sub Saharan African countries, a mean prevalence of 22.4% (SD:9.8%) for S. *haematobium* and 6.21% (SD:5.71%) for HIV was reported [16]. This ecological study further demonstrated that *S*. *haematobium* infection per 100 people was associated with 2.9% relative increase in HIV prevalence (p = 0.038) [16]. Another ecological study based on data from Mozambique, a highly endemic area, reported an association between UGS and HIV/AIDS for all age groups at an odds ratio (OR) of 2.71, 95% CI = 1.56–4.71) [17]. As both studies were based on population level data, both are limited as these conclusions may not be applicable at the individual level. Nevertheless, these studies support the hypothesis that UGS increases susceptibility to HIV acquisition. Some studies, however, found no association between UGS and HIV infection[18,19]. The evidence from epidemiological studies is mixed. In a study in women from northwest Tanzania, female urogenital schistosomiasis was associated with HIV infection at an odds ratio of 4 (95% CI 1.2–26.3) [20]. Another cross-sectional study done in the same area among men reported the odds of HIV infection of 1.3 (95% confidence interval = 0.6–2.5) [18]. This difference in the odds of HIV acquisition by sex is probably due to the anatomic differences in the urogenital systems. A meta-analysis [21] found that HIV-1 shedding in female genital tract was significantly increased in the presence of genital tract infections but little is known about HIV shedding in male genital tract. However, another cross-sectional study conducted in both male and female participants showed no interaction between UGS and HIV/AIDS infection (P = 0.22) [22].

This systematic review and meta-analysis therefore was undertaken to determine if there is an association between UGS and HIV/AIDS infections.

## Methods

### Search strategy

We carried out this systematic review and meta-analysis according to the Centre for Reviews and Dissemination guidelines [23] and the Preferred Reporting Items for Systematic Reviews and Meta-Analyses (PRISMA) statement [24]. The PRISMA Checklist is detailed in S1 Appendix. This review was prospectively registered in PROSPERO database as follows: https://www.crd.york.ac.uk/prospero/display_record.php?ID=CRD42018116648. A protocol exists of this systematic review as detailed in S2 Appendix.

MEDLINE (Ovid), EMBASE (Ovid), Global Health (CABI) (Ovid), and Global Index Medicus were searched for publications before 28^th^ January 2020 with an aim of identifying primary research evidence to answer the research question.

The search strategy was a combination of Medical Subject Heading (MeSH) terms and keywords for Urogenital Schistosomiasis and HIV/AIDS. The search was limited to English language and human subjects. The complete search strategy is detailed in S3 Appendix, S4 Appendix, S5 Appendix, S6 Appendix.

Citation searching was carried out by selecting papers identified for inclusion and then searching for articles that had cited them. Visual scanning of reference lists from relevant studies identified through the database searches was also carried out.

### Inclusion and exclusion criteria

Our initial search included all human studies that assessed the association between UGS and HIV infection. Studies were eligible for inclusion in final analysis if they specifically investigated the association between UGS and HIV infection. UGS and HIV/AIDS infection status should have been laboratory-confirmed. Studies were excluded if they only focused on participants with other pre-existing comorbidities.

### Study selection and data extraction

Two researchers (LZ and FMA) independently screened individual articles by title and abstract, after which fifty-four full text articles were assessed for eligibility for inclusion in this review. Disagreements were resolved by a third reviewer (LY). Thereafter, LZ extracted relevant data from the eligible papers and was cross checked by FMA to ensure consistency. When a potentially relevant publication did not present needed information for meta-analysis, the authors were contacted to request additional data. If the dataset was publicly available, we obtained needed values directly [25].

For each included study, both unadjusted and adjusted odds ratios (OR) were extracted if available. If no unadjusted OR was available in a study, then we calculated the OR by identifying the number of participants in four groups: HIV+ and UGS+; HIV- and UGS+; HIV+ and UGS-; HIV- and UGS-. All data were extracted into an Excel worksheet template. Other variables collected included:

- Bibliographic information
- Study setting
- Study population: age group, sex
- Sample size
- Study design
- Study inclusion and exclusion criteria
- Laboratory test used to confirm Urogenital Schistosomiasis
- Laboratory test used to confirm HIV/AIDS
- Number of participants with UGS and HIV/AIDS
- Number of participants with UGS and without HIV/AIDS
- Number of participants without UGS but with HIV/AIDS
- Number of participants without both UGS and HIV/AIDS
- Unadjusted odds ratio(s), if reported
- If potential confounders were adjusted in the analysis and the adjusted odds ratio(s)
- List of confounders adjusted in the analysis, if applicable

### Statistical analysis

We conducted meta-analyses for unadjusted and adjusted ORs, separately, using random-effects model (REM) by the MetaXL software [26]. REM was used based on the assumption that the true effect of UGS on HIV infection varied from study to study due to differences in mixes in participants secondary to age and social demographics [27]. Due to the limited number of papers that looked at the association of UGS and HIV acquisition in males, meta-analyses were only performed among female-related papers.

### Quality assessment

Study quality was assessed using the Effective Public Health Practice Project (EPHPP) Quality Assessment Tool for Quantitative Studies [28]. This tool checks 6 main components: selection bias, study design, confounders, blinding, data collection method, and withdrawals and dropouts. The tool rates each paper as strong or moderate or weak overall. For sensitivity analysis, any studies with an overall rate of “weak” were excluded from the aforementioned meta-analysis.

## Results

### Search results and study characteristics

**Fig 1:**
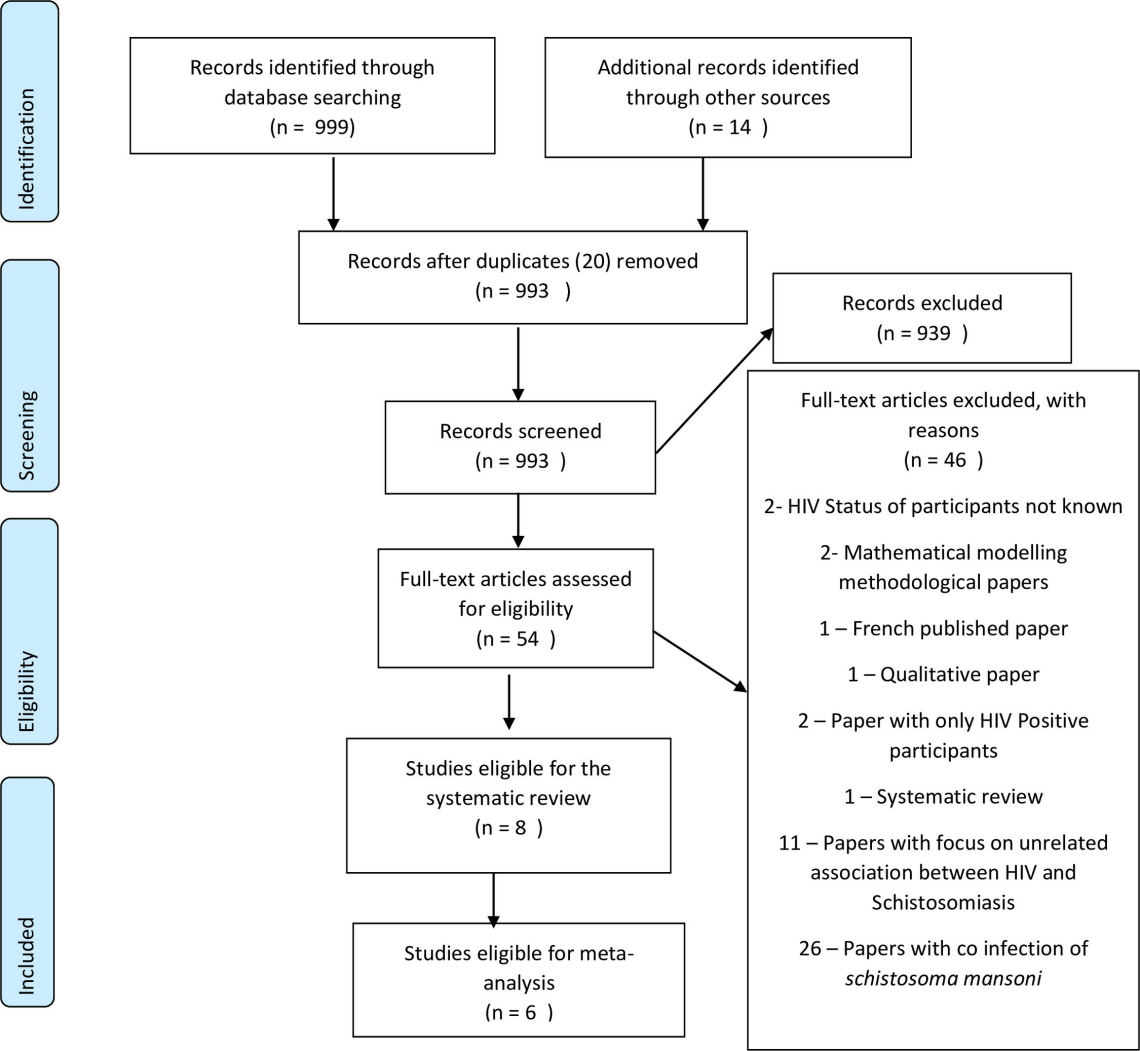
Flow diagram for search and selection of included studies.

A total of 999 studies were obtained from searching four electronic databases (MEDLINE (Ovid) (498), EMBASE (Ovid) (80), Global Health (CABI) (Ovid) (405) and Global Index Medicus (16)). We identified an additional 14 studies through searching the reference lists of potentially eligible studies and consulting a topic expert. The removal of duplicates and screening of titles and abstracts resulted in the exclusion of 939 leaving 54 unique papers for full text review. Of these 54 articles, 46 were excluded with reasons as specified in Fig 1. Eight studies were eligible for inclusion into the systematic review. The PRISMA flow chart below summarises retrieval and selection of the studies [24]. A summary of all the data extracted from the eight included studies can be found in S7 Appendix.

Of the eight included observational studies, seven were cross-sectional studies [18–20,22,29–31] and one was a nested case-control study [25]. All the studies were conducted in Africa–three from Zimbabwe, two from Tanzania, one each from Ghana, South Africa and Zambia. One study [20] was health facility-based and the rest were community-based.

The majority of the included studies (five) were published from the year 2009 onwards, with the largest number (three) having been published between 2015 and 2018. The most recent publication being in 2018. As shown in Table 1, the sample size of the included studies ranged from 331 [30] to 2,145 [25]. Most studies included participants aged 18 years and above. Only one study [29] included participants aged 15 years and above.

**Table 1:** Characteristics of 8 eligible studies included in the systematic review.

Author (Year)	Study design, Setting	N	Age (Years)	Gender	Prevalence of *S*. *haematobium*	Prevalence of HIV	UGS confirmatory Laboratory testing	HIV confirmatory laboratory testing
Kallestrup et al (2005)	Cross-sectional, Rural Zimbabwe	1545	>18	Men and women	43.4%	26.3%	Microscopic examination of fixed volume urine samples filtered on Nytrel filters	Rapid HIV-1/2 test kit (Determine) and confirmation was done with Oraquick or Capillus.
Kjetland et al (2006)	Cross-sectional, Rural Zimbabwe	445	20–49	Women	46%	29%	Urine samples examined for *S*. *haematobium* ova. A single terminal-spined ovum gave a positive diagnosis in Pap smears, wet mounts or biopsies of genital tissue	HIV 1 serological tests
Ndhlovu et al, (2007)	Cross-sectional, Rural Zimbabwe	544	15–49	women	40%	29%	urine samples collected on three consecutive days. Urine specimens examined by the filtration technique	Genelavia Mixt HIV-1/2 ELISA. The second test was the Recombigen HIV-1/2 enzyme immunoassay
Yirenya—Tawiah et al (2009)	Cross-sectional, Ghana (Rural—Volta Basin)	331	20–49	Women	10.3%	7.9%	At least one *schistosome* ovum detected in cervical biopsy tissue	Determine HIV—1/2 Test and an immunochromatographic test
Downs et al (2011)	Cross-sectional, Rural Tanzania	457	18–50	Women	5%	5.9%	A single urine sample filtered and examined microscopically for *Schistosoma* ova. Abnormal cervical lesions biopsied. Specimens stained with Hematoxylin and Eosin (H&E) for histopathological examination and Trypan Blue to examine for *Schistosoma* ova using the crush technique	Rapid test (SD Bioline)
Kleppa et al (2015)	Cross-sectional, Urban South Africa	752	>16	Women	31%	16.1%	*Schistosoma* eggs in urine counted by microscopy	Bioline Rapid Test HIV and confirmatory Sensa Tri- Line HIV Test Kit.
Downs et al (2017)	Cross-sectional, Rural Tanzania	674	18–50	Men	53.6%	6.3%	*S*. *haematobium* ova in urine and/or OR CAA 30 pg/mL in an *S*. *haematobium* endemic region in an individual with no S. mansoni ova in stool.	Rapid tests (SD Bioline) were used with confirmatory testing for positive samples (Unigold) as per the national testing algorithm.
Wall et al (2018)	Nested case-control, Rural Zambia	2145	>18	Men and women	59%	55.7%	A positive SWAP antibody response. Immunoblot testing using species-specific antigens to distinguish between *S*. *mansoni* and *S*. *haematobium* antibodies	Rapid HIV antibody testing, ELISA and RNA polymerase chain reaction.

### Quality assessment of the studies

The quality assessment was done using the Effective Public Health Practice Project (EPHPP) Quality Assessment Tool for Quantitative Studies [28]. Of the eight papers included the overall rating of each paper was as shown in S8 Appendix. Overall, seven of the eight papers had moderate to strong quality assessment ratings.

### Association between UGS and HIV infection outcome

All studies assessed the association between UGS and HIV/AIDS. The unadjusted OR across all the eight papers ranged from 0.82 to 3.8 as summarised in Table 2 below.

**Table 2:** Summary of unadjusted OR and quality score of the 8 shortlisted papers.

Study	Gender	Unadjusted OR (95% CI)	Number of participants	Quality Assessment
Kallestrup et al (2005) [22]	Male and females	0.96 (0.75–1.24)	1545	Moderate
Kjetland et al (2006) [29]	Female	2.06 (1.21–3.49)	445	Strong
Ndhlovu et al, (2007) [31]	Female	1.45 (1–2.12)	544	Moderate
Yirenya—Tawiah et al (2009) [30]	Female	1.42 (0.40–5.06)	331	Weak
Downs et al (2011) [20]	Female	3.76 (1.18–11.97)	457	Moderate
Kleppa et al (2015) [19]	Female	0.83 (0.5–1.39)	752	Moderate
Downs et al (2017) [18]	Male	1.41 (0.60–3.29)	674	Strong
Wall et al (2018) [25]	Male	0.78 (0.6–1.01)	1046	Moderate
Wall et al (2018) [25]	Female	0.82 (0.65–1.04)	1099	Moderate

**Fig 2:**
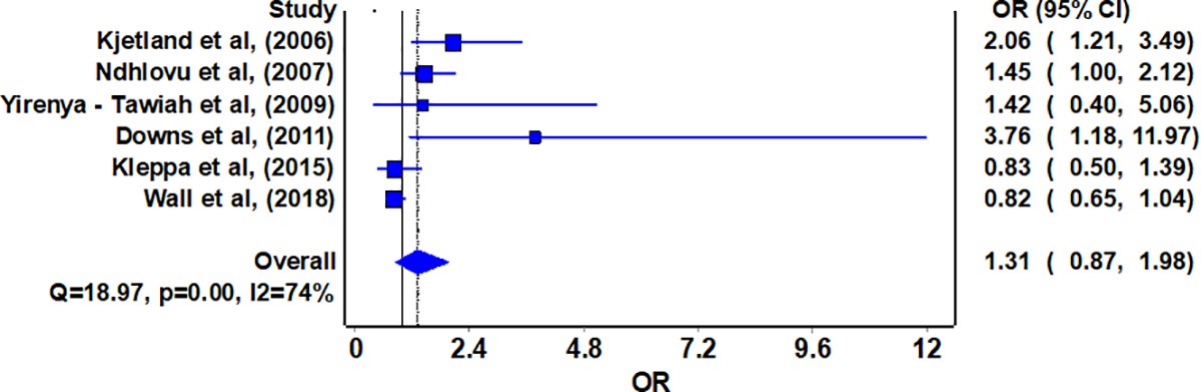
Forest plot of impact of Urogenital schistosome infection to HIV infection in women.

Of the eight studies, five studies reported the unadjusted OR between UGS and HIV infection in females [19,20,29–31].As shown in Fig 2, pooled estimate from the unadjusted ORs showed that female UGS was not associated with HIV infection (OR 1.31, 95% CI 0.87–1.99). Similar estimate was observed in our sensitivity analysis excluding one study with an overall rate of “weak” (OR 1.32, 95% CI 0.83–2.10).

Of the four studies that reported an adjusted OR as shown in the Table 3, three were done in participants who were only women and one in which participants were both men and women.

**Table 3:** Summary of the shortlisted papers that reported an adjusted OR.

Study	Gender	Adjusted OR (95% CI)	Variables adjusted for
Kjetland et., al (2006)	Women	2.1 (1.2–3.5)	Age and BMI
Ndhlovu et., al (2007)	Women	1.4 (0.93–2.0)	Tribal origin, urban childhood and Age
Downs et., al (2011)	Women	4.0 (1.2–13.5)	Age, Gynaecological symptoms and all baseline characteristics (Marital status, number of children, people living in household, occupation, went to bed hungry, number of water contacts per day, ever treated for schistosomiasis, received artemesinin medication in past 3 years for malaria).
Downs et., al (2017)	Men	1.4 (0.6–3.3)	Age, Years of school completed, number of sexual partners in the past 6 months, dyspareunia, number of people living in household, age in years after first sex, typical sex partners more than 5 years younger, ever treated for STI, hemospermia, painful genital ulcers, syphilis.

**Fig 3:**
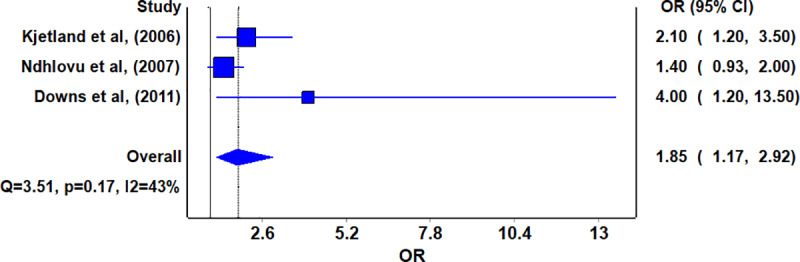
Forest plot of impact of Urogenital schistosome infection to HIV infection in papers with adjusted OR.

Further meta-analysis of papers that reported adjusted OR in women was done as shown in Fig 3 below.

Pooled effects of the three studies showed that female UGS was associated with HIV infection (OR 1.85, 95% CI 1.17–2.92).

## Discussion

This systematic review and meta-analysis assessing the association between UGS and HIV/AIDS infection supports the hypothesis that there could be an association between these two infections in females. In males, association could not be ascertained due to insufficient evidence.

According to our knowledge, this is the first systematic review with a meta-analysis to explore the association between urogenital schistosomiasis and HIV/AIDS infection. Little research has been done on this topic and for the studies done, a majority of them are not prospective studies. In a recently published systematic review that explored the interaction between all species of *Schistosoma* and HIV, it was concluded that *S*. *haematobium* increased the risk of HIV acquisition [32]. This systematic review, however, did not report specifically the effect of *S*. *haematobium* infection and was limited by the lack of a meta-analysis. Other [33,34]systematic reviews done have explored the association between helminths (including schistosomiasis but not specifically UGS) and HIV infection and most of them explored the effect of treatment with praziquantel on HIV progression. A recently conducted Cochrane review that evaluated the effects of antihelminthics (albendazole and praziquantel) on markers of HIV disease progression, concluded that treating helminth infections including schistosomiasis may have a small suppressive effect on HIV viral load [33]. This finding was similar to one from an earlier systematic review that focused on the effect of treating co-infections (tuberculosis, malaria, geohelminthiasis, schistosomiasis and sexually transmitted infections) on HIV viral load. It showed that standardized mean plasma viral load decreased after the treatment of the coinfections [34]. It is mindful to note that in that systematic review, of the 18 papers eligible, only one was on schistosomiasis. So this beneficial effect would have been due to the other infections but not necessarily schistosomiasis.

Another earlier systematic review was inconclusive due to insufficient data available. The authors recommended more research is needed but highlighted a single randomised clinical trial (RCT) and multiple observational studies that suggested a possible benefit of helminth eradication in reducing HIV viral load [35]. A reduction of viral load is posited to reduce risk of HIV transmission.

Overall, although meta-analysis results with papers reporting unadjusted OR point to no likely association between UGS and HIV infections in women (OR 1.31, 95% CI 0.87–1.99), meta-analysis done with studies that reported adjusted OR, shows an association between UGS and HIV/AIDS infections (OR 1.85, 95% CI 1.17–2.92). Adjusted ORs were used as they controlled for confounders like age and occupation during analysis that would have led to an alternative explanation for an association between UGS and HIV/AIDS infections.

Although our findings could not indicate the direction of the observed association between UGS and HIV infection among females, there is evidence in the literature affirming the biological plausibility that genital inflammation increases HIV infection susceptibility. In a phase 2b double blind RCT done in South African women, it was shown that genital inflammation was associated with increased risk of HIV acquisition (odds ratio, 3.2; 95% CI, 1.3–7.9; P = 0.014) [36]. Although this study did not focus on UGS but generally on the presence of genital inflammation, it affirms the thinking that this inflammation secondary to diseases like UGS, could lead to increased HIV acquisition. Our finding that *S*. *haematobium* is associated with HIV, are similar to current evidence pointing an association between *S*. *mansoni* infection and HIV infection [37].

For policy, our investigation suggests a likely association between UGS and HIV/AIDS in women. This review provides evidence that there is need for evidence-based policies aimed at improving and supporting research about neglected tropical diseases (NTDs) like schistosomiasis. These policies should be formulated and implemented as part of wider policies aimed at eliminating schistosomiasis as recommended by WHO in the sixty-fifth world health assembly held in 2012 [38].

For practice, this review cannot establish a causal relationship between UGS and HIV infection. Thus, changes to practice will not be suggested based on findings of this review.

For research, large-scale prospective studies are needed in future to investigate this association further. For the research currently done, all the studies had small sample sizes as evidenced from the ones included. It is mindful to remember that ethical issues have been highlighted concerning research on the association between UGS and HIV infection. In women, issues of doing colposcopy in virgins and exposing them to infections by undertaking biopsies have made this type of research delicate and difficult [3]. WHO hence recommends that whatever research is done, it should be culture sensitive and able to maintain the sensitive balance between the risks and the benefits of the study population. Evidence in this systematic review is from a few endemic countries such as Zambia, Zimbabwe and South Africa. This might not be representative for all UGS endemic countries in SSA. There is need for more research in other countries to elucidate further on the association or even better on the casual relationship between UGS and HIV/AIDS. As meta-analysis was only conducted for female studies, the results of this systematic review do not provide complete evidence for any association between UGS and HIV infection in males. There are limited studies looking at UGS in men and HIV. In a systematic review done on MGS, only 31 research articles were identified that exclusively focused on MGS [5]. Another systematic review on MGS identified 40 publications that were dominated by case reports and observational studies that focused on MGS [39]. Future meta-analysis is recommended as more primary research involving males becomes available.

Among the study’s limitations, chance could have influenced some of the study findings. Formal sample size calculation was only carried out in two of the eight included studies [18,19].

Selection bias may have occurred if the selected participants were not representative of the target population. Of the eight studies, only one [20] involved recruiting research participants at the health facility. For the remainder, recruitment was community-based. This may have reduced selection bias overall as participants of the seven studies were representative of the target population compared to that one study. None of the eight studies reported using any random sampling techniques, a limitation that could have introduced selection bias.

Measurement bias could have resulted from the way UGS and HIV infection were diagnosed in all the included studies. All eight reported which laboratory test was used to define UGS infection. Seven of the eight studies diagnosed UGS by identifying *S*. *haematobium* ova and one [25] used an antibody response to *Schistosom*a and proceeded further to distinguish between *S*. *mansoni* and *S*. *haematobium*. Though these are all recommended tests for diagnosing UGS, this highlights the lack of a gold standard testing for *S*. *haematobium* [40]. Furthermore, women have been shown to have lower odds of egg secretion than men (OR = 0.4 (0.3,0.5), P<0.001) which could possibly have affected the results [41]. For HIV infection, all the eight studies used serological diagnostic tests, in line with WHO recommendations of HIV laboratory diagnosis. In addition, not all studies controlled for age and baseline characteristics that were statistically significant. Only two controlled for these baseline characteristics during analysis. [18,25]. Other confounders such as occupation were only considered in one study [20]. Overall, though these limitations could have affected our meta-analysis, they are not direct limitations of our study.

This systematic review was only able to conduct a meta-analysis in females. Due to insufficient evidence in males, a meta-analysis could not be conducted. Furthermore, as the number of studies that reported adjusted OR was not sufficient to assess publication bias, we cannot rule out publication bias, as studies reporting positive association are potentially more favourable for publication than those reporting no association.

The findings of this research may prove useful in expanding the understanding of the effect of UGS on the trajectory of HIV infection in SSA. In such settings, where the burden of both infections is high, this understanding may offer an entry point in the implementation of low-cost public health interventions in the prevention and control of the HIV epidemic.

## Supporting information

S1 Appendix The PRISMA 2009 checklist. (DOC)

S2 Appendix The published systematic review protocol. (DOCX)

S3 Appendix The search strategy for EMBASE database. (DOCX)

S4 Appendix The search strategy for MEDLINE database. (DOCX)

S5 Appendix The search strategy for Global Health (CABI) database. (DOCX)

S6 Appendix The search strategy for Global Index Medicus database. (DOCX)

S7 Appendix The data extraction tool with all the included studies. (DOCX)

S8 Appendix The Quality Assessment scores of the included studies. (DOCX)
