# Supplementary material for: A systematic review and meta-analysis to assess the association between urogenital schistosomiasis and HIV/AIDS infection
Source: PLoS Negl Trop Dis. 2020 Jun 15;14(6):e0008383. doi: 10.1371/journal.pntd.0008383 (PMC7316344; doi:10.1371/journal.pntd.0008383)
Supplement: S2 Appendix — (DOCX) [file pntd.0008383.s002.docx]

Title

A systematic review protocol to assess the association between Human urogenital schistosomiasis and HIV infection.

Background

Schistosomiasis a neglected parasitic worm infection is a disease of public health importance with a high mortality of 150,000 to 300, 000 per year in sub Saharan Africa attributed to schistosoma mansoni and schistosoma haematobium(van der Werf *et al.*, 2003).

192 million (93%) of the world’s estimated 207 million cases of schistosomiasis occur in sub Saharan Africa(Hotez and Kamath, 2009). Furthermore, 85% of the neglected tropical disease burden in sub Saharan Africa is due to helminth infections with the second most prevalent among them being schistosomiasis(Hotez and Kamath, 2009), the most prevalent type being Schistosomiasis Mansoni(Steinmann *et al.*, 2006).

HIV prevalence is high in communities with a high burden of genital Schistosomiasis(Eyrun F Kjetland *et al.*, 2014). Through observational studies, an association has been shown between the high prevalence of both genital schistosomiasis and HIV(Mbah *et al.*, 2013).

Through other observational studies, the association between urogenital schistosomiasis and HIV infection has been found to be due to urogenital schistosomiasis’ ability to facilitate the uptake of HIV virus by increasing HIV target cells like CD 4+ T cells(Kleppa *et al.*, 2014). It has been further shown that urogenital schistosomiasis results in increased genital vascularity which can result in HIV acquisition(Eyrun F. Kjetland *et al.*, 2014). This increased odds of acquiring HIV has not only been associated with Schistosomiasis haematobium but as well as with schistosomiasis mansoni(Jennifer A. Downs *et al.*, 2017).

Schistosomiasis has further been found to harm humans by eliciting immune responses that affect ways in which the body responds to other infections(Taylor-Robinson, Jones and Garner, 2007). In Uganda, it has been associated with increased susceptibility to HIV at a population level in fishing communities (Sanya *et al.*, 2015). Though this association was made, Sanya et al (2015) concluded that at an individual level, schistosomiasis mansoni is not an important risk factor for HIV a conclusion similar to another nested case control study done in Uganda that showed that Schistosomiasis was not associated with HIV acquisition(Ssetaala *et al.*, 2015). The same conclusion was done on the Tanzania side in men as schistosome infection was not found to have a higher odds of HIV infection when compared to those without schistosome infection(Jennifer A Downs *et al.*, 2017). Though this was concluded, in another study it was concluded that women had an increased prevalence of HIV infection (Downs *et al.*, 2012). The difference between the Tanzanian and Ugandan studies is that the former focused on schistosomiasis caused by both schistosoma mansoni and Haematobium whereas the later was only on that infection due to schistosoma mansoni.

As all these studies where observational studies, a systematic review is therefore warranted to combine all the available studies because of the existing high burden of both Schistosomiasis and HIV infections within the same communities as an understanding of this will be critical in informing HIV prevention strategies.

Research question

“What is the association between Urogenital Schistosomiasis and HIV infection as assessed by HIV clinical presentation and immunological responses?”

Population and Intervention: Adults with confirmed Schistosomiasis haematobium.

Control: Adults without confirmed Schistosomiasis.

Outcome: Positive HIV antigen/antibody test; HIV Viral load and clinical symptoms of HIV/AIDS.

Description of the methodology

**Identification of research evidence**

Different approaches will be used namely:

- Searching electronic databases like the MEDLINE, EMBASE, Global Health (CABI), and Global Index Medicus (<http://www.globalhealthlibrary.net/php/index.php>)
- Visually scanning reference lists from relevant studies identified through the database searches.
- Hand searching key journals identified from results of the data base searches to identify those that contain the largest number of relevant studies.
- Topic expects in the area of tropical health and Infectious diseases will be asked to check the list of shortlisted relevant studies to identify any known missing studies not included.
- Citation searching, this will involve selecting a number of key papers already identified for inclusion and then searching for articles that have cited them.

**Inclusion criteria**

1. Randomized controlled trials (RCTs), cohort, case controls and cross sectional study designs.
2. Studies conducted in human participants.
3. Studies in which participants’ Urogenital Schistosoma infection status was laboratory confirmed by direct urine microscopy, concentration techniques, other microscopic methods), culture of urine samples, antigen testing methods (e.g. Circulating cathodic antigen (CCA)and Circulating anodic antigen (CAA) tests) and antibody based detection using Enzyme linked Immunosorbent assay (ELISA).
4. Studies in which HIV infection status was decided by clinical presentation, immunological responses, and/or laboratory evidence.
5. Studies published in English.
6. Studies published in peer reviewed journals.

**Exclusion criteria**

1. Case series and case reports.
2. Studies with Other preexisting comorbidities

**Search strategy**

Humans AND (Trematode OR Schistosoma OR Haematobium OR genital disease OR lower reproductive tract OR MGS OR FGS) AND (positive HIV sero status OR Plasma HIV viral load OR Clinical HIV Presentation OR HIV OR AIDS OR HIV/AIDS)

This search strategy will be refined and finalized with the assistance of the university librarian and will focus on literature within the period up to October 2018.

The bibliographic software Mendeley will be used to record and manage references.

**Study selection**

The selection will be done by first screening all papers using their titles and abstracts and later screening of the full papers that meet the inclusion criteria for which a definite decision cannot be made based on the title and abstract alone(CRD, 2009).

This process will be summarized with a flow chart as guided by the PRISMA guidelines(Liberati *et al.*, 2009).

**Data extraction**

A sample data extraction form will have the following items:

- Date of data extraction
- Identification features of the study:
  - Record number (to uniquely identify study)
  - Author
  - Article title
  - Citation
  - Type of publication (e.g. journal article)
  - Country of origin
  - Funding source
- Study characteristics
  - Aim/objectives of the study
  - Study design
  - Study inclusion and exclusion criteria
  - Recruitment procedures used (e.g. details of randomisation, blinding)
- Participant characteristics
- Characteristics of participants at the beginning of the study e.g.
  - Age
  - Gender
  - Ethnicity
  - Occupation
  - Marital status
  - Number of lifetime sexual partners
- Intervention details
  - Type of laboratory test used to confirm genital Schistosomiasis infection
  - The Laboratory test used to confirm HIV infection
- Outcome data/results
  - Statistical techniques used
  - For each outcome:
    - Whether reported
    - Definition used in study
    - Measurement tool or method used
    - Unit of measurement (if appropriate)
    - Length of follow-up, number and/or times of follow-up measurements

For all study group(s):

- Number of participants enrolled
- Number of participants included in analysis
- Number of withdrawals, exclusions, lost to follow-up
- Summary outcome data e.g.
  - Dichotomous: number of events, number of participants
  - Continuous: mean and standard deviation

Type of analysis used in study (e.g. intention to treat, per protocol)

Results of study analysis e.g.

- Dichotomous: odds ratio, risk ratio and confidence intervals, p-value
- Continuous: mean difference, confidence intervals

**Quality assessment**

To ensure quality, appropriateness of study design and critical review of individual aspects of it that may introduce bias will be done guided by CASP checklists(Singh, 2013).

**Data synthesis**

Depending on the nature of data collated, combined and summarized, formal statistical quantitative techniques such as meta-analysis will be done or if formal pooling of result is in appropriate, a narrative approach will be done.

**Dissemination of findings**

A planned and active process to disseminate the results will be used. Within my workplace, team presentations will be done together with local and foreign conference presentations. The final manuscript will be submitted for publication in a peer reviewed journal.

Assessment of potential ethical or other risks, limitations and/or difficulties

Level 1 form will be submitted to the usher Institute Ethics Committee.

A risk of loss of data collected is possible in cases where it is corrupted by a virus or when the laptop or storage unit is lost / stolen. To minimize such losses, all data will be backed up online in drop box daily. As well an up to date anti-virus will be installed on the laptop or gadget to be used.

The limitation of bad internet connection is foreseen. This will be countered by having access to at least 2 internet providers so that one acts as a backup.

The other limitation is time; this will be addressed by having a well laid out plan with timelines agreed with my supervisors.

The difficulty of accessing articles in full text is foreseen as payment will be required for some. This will be addressed by using the University of Edinburgh DiscoverEd library portal and when this fails to consult the librarian so that a full text is provided.

Outline of milestones and their delivery times throughout the duration of the project

| **Thing to do** | **University Deadline** | **When to complete task** |
| --- | --- | --- |
| **Dissertation** | **3rd June 2019** |  |
| Refine and finalise the search strategy to use with Supervisor and University Librarian |  | 30^th^ November 2018 |
| Submit Level 1 UoE ethics application | **5^th^ November 2018** | 5^th^ November 2018 |
| Carry out search on the selected Electronic literature data bases |  | 30^th^ November 2018 |
| Import the search results into Mendeley |  | 30^th^ November 2018 |
| Title and Abstract screening and other identified sources |  | 15^th^ December 2018 |
| Full text screening and selection of the selected and agreed relevant records |  | 15^th^ December 2019 |
| Extract data from all selected full-text articles |  | 22^nd^ December 2019 |
| Assess the quality of the articles identified using an agreed quality appraisal tool |  | 15^th^ January 2019 |
| Data Synthesis and interpretation |  | 10^th^ Feb 2019 |
| Submit in the draft chapter of result to supervisor for feedback |  | 28^th^ Feb 2019 |
| Submit in the draft chapter of literature review to supervisor for feedback |  | 15^th^ March 2019 |
| Submit in the draft chapter of research design and method to supervisor for feedback |  | 29^th^ March 2019 |
| Submit in the draft chapters on discussion and conclusion to supervisor for feedback |  | 25^th^ April 2019 |
| Submit full dissertation draft to supervisor for feedback | **7^th^ May 2019** | **5^th^ May 2019** |
| Respond to queries from supervisor and re-share for second review |  | 21^st^ May 2019 |
| Submit final dissertation and reflective review to Turnitin | **3^rd^ June 2019** | **2^nd^ June 2019** |

**References**

CRD (2009) *Sytematic Reviews*. York: CRD, University of York.

Downs, J. A. *et al.* (2012) ‘Association of Schistosomiasis and HIV infection in Tanzania.’, *The American journal of tropical medicine and hygiene*. United States, 87(5), pp. 868–873. doi: https://dx.doi.org/10.4269/ajtmh.2012.12-0395.

Downs, J. A. *et al.* (2017) ‘Effects of schistosomiasis on susceptibility to HIV-1 infection and HIV-1 viral load at HIV-1 seroconversion: A nested case-control study’, *PLOS Neglected Tropical Diseases*. Edited by M. H. Hsieh. Public Library of Science, 11(9), p. e0005968. doi: 10.1371/journal.pntd.0005968.

Downs, J. A. *et al.* (2017) ‘Schistosomiasis and Human Immunodeficiency Virus in Men in Tanzania.’, *The American journal of tropical medicine and hygiene*. United States, 96(4), pp. 856–862. doi: https://dx.doi.org/10.4269/ajtmh.16-0897.

Hotez, P. J. and Kamath, A. (2009) ‘Neglected Tropical Diseases in Sub-Saharan Africa: Review of Their Prevalence, Distribution, and Disease Burden’, *PLoS Neglected Tropical Diseases*. Edited by M. Cappello. Public Library of Science, 3(8), p. e412. doi: 10.1371/journal.pntd.0000412.

Kjetland, E. F. *et al.* (2014) ‘Classification of the lesions observed in female genital schistosomiasis’, *International Journal of Gynecology & Obstetrics*. Wiley-Blackwell, 127(3), pp. 227–228. doi: 10.1016/j.ijgo.2014.07.014.

Kjetland, E. F. *et al.* (2014) ‘Genital schistosomiasis and its unacknowledged role on HIV transmission in the STD intervention studies’, *International Journal of STD & AIDS*. SAGE PublicationsSage UK: London, England, 25(10), pp. 705–715. doi: 10.1177/0956462414523743.

Kleppa, E. *et al.* (2014) ‘Effect of Female Genital Schistosomiasis and Anti-Schistosomal Treatment on Monocytes, CD4+ T-Cells and CCR5 Expression in the Female Genital Tract’, *PLoS ONE*. Edited by A. J. F. LUTY. Public Library of Science, 9(6), p. e98593. doi: 10.1371/journal.pone.0098593.

Liberati, A. *et al.* (2009) ‘The PRISMA Statement for Reporting Systematic Reviews and Meta-Analyses of Studies That Evaluate Health Care Interventions: Explanation and Elaboration’, *PLoS Medicine*. Public Library of Science, 6(7), p. e1000100. doi: 10.1371/journal.pmed.1000100.

Mbah, M. L. N. *et al.* (2013) ‘HIV and Schistosoma haematobium prevalences correlate in sub-Saharan Africa.’, *Tropical medicine & international health : TM & IH*. England, 18(10), pp. 1174–1179. doi: https://dx.doi.org/10.1111/tmi.12165.

Sanya, R. E. *et al.* (2015) ‘Schistosoma mansoni and HIV infection in a Ugandan population with high HIV and helminth prevalence’, *Tropical Medicine & International Health*. Wiley/Blackwell (10.1111), 20(9), pp. 1201–1208. doi: 10.1111/tmi.12545.

Singh, J. (2013) ‘Critical appraisal skills programme’, *Journal of Pharmacology and Pharmacotherapeutics*. Medknow Publications and Media Pvt. Ltd., 4(1), p. 76. doi: 10.4103/0976-500X.107697.

Ssetaala, A. *et al.* (2015) ‘Schistosoma mansoni and HIV acquisition in fishing communities of Lake Victoria, Uganda: a nested case-control study’, *Tropical Medicine & International Health*. Wiley/Blackwell (10.1111), 20(9), pp. 1190–1195. doi: 10.1111/tmi.12531.

Steinmann, P. *et al.* (2006) ‘Schistosomiasis and water resources development: systematic review, meta-analysis, and estimates of people at risk’, *The Lancet Infectious Diseases*. Elsevier, 6(7), pp. 411–425. doi: 10.1016/S1473-3099(06)70521-7.

Taylor-Robinson, D. C., Jones, A. P. and Garner, P. (2007) ‘Deworming drugs for treating soil-transmitted intestinal worms in children: effects on growth and school performance’, in Taylor-Robinson, D. C. (ed.) *Cochrane Database of Systematic Reviews*. Chichester, UK: John Wiley & Sons, Ltd. doi: 10.1002/14651858.CD000371.pub3.

van der Werf, M. J. *et al.* (2003) ‘Quantification of clinical morbidity associated with schistosome infection in sub-Saharan Africa’, *Acta Tropica*. Elsevier, 86(2–3), pp. 125–139. doi: 10.1016/S0001-706X(03)00029-9.
