# Supplementary material for: A systematic review and meta-analysis to assess the association between urogenital schistosomiasis and HIV/AIDS infection
Source: PLoS Negl Trop Dis. 2020 Jun 15;14(6):e0008383. doi: 10.1371/journal.pntd.0008383 (PMC7316344; doi:10.1371/journal.pntd.0008383)
Supplement: S3 Appendix — (DOCX) [file pntd.0008383.s003.docx]

| 1 | exp Human immunodeficiency virus/ | 179680 |
| --- | --- | --- |
| 2 | exp Human immunodeficiency virus 1/ | 74615 |
| 3 | exp Human immunodeficiency virus infection/ | 354971 |
| 4 | (human immun* adj1 virus).mp. [mp=title, abstract, heading word, drug trade name, original title, device manufacturer, drug manufacturer, device trade name, keyword, floating subheading word, candidate term word] | 410363 |
| 5 | exp acquired immune deficiency syndrome/ | 137832 |
| 6 | (acquired immun* adj1 syndrom*).mp. [mp=title, abstract, heading word, drug trade name, original title, device manufacturer, drug manufacturer, device trade name, keyword, floating subheading word, candidate term word] | 21583 |
| 7 | 1 or 2 or 3 or 4 or 5 or 6 | 473278 |
| 8 | female genital tract infection/ or exp schistosomiasis/ or exp schistosomiasis haematobia/ or exp Schistosoma hematobium/ | 26890 |
| 9 | schistosom*.ti,ab. | 33466 |
| 10 | 8 or 9 | 40920 |
| 11 | 7 and 10 | 1127 |
| 12 | limit 11 to (outcomes research and english and article and journal) | 0 |
| 13 | limit 11 to journal | 1115 |
| 14 | limit 13 to english | 1059 |
| 15 | limit 14 to human | 943 |
| 16 | limit 15 to exclude medline journals | 67 |
