# Supplementary material for: A systematic review and meta-analysis to assess the association between urogenital schistosomiasis and HIV/AIDS infection
Source: PLoS Negl Trop Dis. 2020 Jun 15;14(6):e0008383. doi: 10.1371/journal.pntd.0008383 (PMC7316344; doi:10.1371/journal.pntd.0008383)
Supplement: S4 Appendix — (DOCX) [file pntd.0008383.s004.docx]

| 1 | HIV/ or exp HIV-1/ |
| --- | --- |
| 2 | exp HIV Infections/ |
| 3 | (human immun* adj1 virus).mp. [mp=title, abstract, original title, name of substance word, subject heading word, floating sub-heading word, keyword heading word, protocol supplementary concept word, rare disease supplementary concept word, unique identifier, synonyms] |
| 4 | exp Acquired Immunodeficiency Syndrome/ |
| 5 | 1 or 2 or 3 or 4 |
| 6 | Genital Diseases, Female/ or exp Schistosomiasis haematobia/ or exp Schistosoma haematobium/ or Schistosomiasis/ |
| 7 | exp SCHISTOSOMIASIS HAEMATOBIA/ or SCHISTOSOMIASIS/ |
| 8 | schistosom*.ti,ab. |
| 9 | 6 or 7 or 8 |
| 10 | 5 and 9 |
| 11 | limit 10 to (english and (adaptive clinical trial or clinical study or clinical trial or comparative study or controlled clinical trial or equivalence trial or observational study or pragmatic clinical trial or randomized controlled trial)) |
| 12 | limit 10 to (humans and english) |
| 13 | limit 12 to (observational study or randomized controlled trial) |
| 14 | limit 12 to journal article |

S1 Appendix: Search Strategy for MEDLINE (Ovid)
