# Supplementary material for: A systematic review and meta-analysis to assess the association between urogenital schistosomiasis and HIV/AIDS infection
Source: PLoS Negl Trop Dis. 2020 Jun 15;14(6):e0008383. doi: 10.1371/journal.pntd.0008383 (PMC7316344; doi:10.1371/journal.pntd.0008383)
Supplement: S5 Appendix — (DOCX) [file pntd.0008383.s005.docx]

| 1 | HIV.mp. [mp=abstract, title, original title, broad terms, heading words, identifiers, cabicodes] | 164187 |
| --- | --- | --- |
| 2 | HIV-1.mp. [mp=abstract, title, original title, broad terms, heading words, identifiers, cabicodes] | 32828 |
| 3 | HIV infections.mp. [mp=abstract, title, original title, broad terms, heading words, identifiers, cabicodes] | 118564 |
| 4 | (human immun* adj1 virus).mp. [mp=abstract, title, original title, broad terms, heading words, identifiers, cabicodes] | 157391 |
| 5 | Acquired Immunodeficiency Syndrome.mp. [mp=abstract, title, original title, broad terms, heading words, identifiers, cabicodes] | 4727 |
| 6 | acquired immun*adj1 syndrom*.mp. [mp=abstract, title, original title, broad terms, heading words, identifiers, cabicodes] | 0 |
| 7 | 1 or 2 or 3 or 4 or 5 | 173046 |
| 8 | Genital Schistosomiasis.mp. [mp=abstract, title, original title, broad terms, heading words, identifiers, cabicodes] | 122 |
| 9 | Schistosomiasis.mp. [mp=abstract, title, original title, broad terms, heading words, identifiers, cabicodes] | 46527 |
| 10 | SCHISTOSOMIASIS HAEMATOBIA.mp. [mp=abstract, title, original title, broad terms, heading words, identifiers, cabicodes] | 160 |
| 11 | Schistosoma haematobium.mp. [mp=abstract, title, original title, broad terms, heading words, identifiers, cabicodes] | 7654 |
| 12 | schistosom*.ti,ab. | 47728 |
| 13 | 8 or 9 or 10 or 11 or 12 | 51138 |
| 14 | 7 and 13 | 480 |
| 15 | limit 14 to (english and journal article) | 376 |
