# Supplementary material for: A systematic review and meta-analysis to assess the association between urogenital schistosomiasis and HIV/AIDS infection
Source: PLoS Negl Trop Dis. 2020 Jun 15;14(6):e0008383. doi: 10.1371/journal.pntd.0008383 (PMC7316344; doi:10.1371/journal.pntd.0008383)
Supplement: S6 Appendix — (DOCX) [file pntd.0008383.s006.docx]

| tw:((tw:(hiv)) OR (tw:(hiv - 1)) OR (tw:(aids)) OR (tw:(acquired immunodeficiency syndrome)) AND (tw:(genital schistosomiasis)) OR (tw:(schistosomiasis haematobia)) OR (tw:(schistosoma haematobium)) AND (tw:(schistosomiasis))) AND (instance:"ghl") |
| --- |
| - Limits   - Humans [(remover)](javascript:remove_filter('limit_humans'))   - Female [(remover)](javascript:remove_filter('limit_female'))   - Adult [(remover)](javascript:remove_filter('limit_adult'))   - Adolescent [(remover)](javascript:remove_filter('limit_adolescent'))   - Young_adult [(remover)](javascript:remove_filter('limit_young-adult'))   - Middle aged [(remover)](javascript:remove_filter('limit_middle-aged'))   - Male [(remover)](javascript:remove_filter('limit_male'))   - Child [(remover)](javascript:remove_filter('limit_child'))   - Pregnancy [(remover)](javascript:remove_filter('limit_pregnancy')) - Language   - English [(remover)](javascript:remove_filter('la_en'))   [Home](http://www.globalhealthlibrary.net/?lang=en) > [Search](http://search.bvsalud.org/ghl/?lang=en) > (tw:(Genital Schistosomiasis)) AND (tw:(HIV )) (41) |
