# Supplementary material for: A systematic review and meta-analysis to assess the association between urogenital schistosomiasis and HIV/AIDS infection
Source: PLoS Negl Trop Dis. 2020 Jun 15;14(6):e0008383. doi: 10.1371/journal.pntd.0008383 (PMC7316344; doi:10.1371/journal.pntd.0008383)
Supplement: S7 Appendix — (DOCX) [file pntd.0008383.s007.docx]

| **Author (Year of publication)** | **Article title** | **Year of study** | **Study design** | **study setting** | **Number of participants** | **Sex** | **Age** | **Incl & Excl Criteria** | **Laboratory Test used to confirm GS** | **Laboratory test used to Confirm HIV (CD4 Count if provided)** | **FGS+ & HIV+** | **FGS- & HIV+** | **FGS+ & HIV-** | **FGS- & HIV-** | **Measure of association OR / P Value / CI** (CALCULATED) | **Confounders identified by authors** | **Notes** |
| --- | --- | --- | --- | --- | --- | --- | --- | --- | --- | --- | --- | --- | --- | --- | --- | --- | --- |
| Yirenya - Tawiah et al (2009) | HIV testing in community based research a case study of female genital schistosomiasis and HIV in the Volta Basin of Ghana | Not Mentioned | Cross sectional | Ghana (Rural - Volta Basin) | 331 | Female | 20-49 | Inclusion criteria:Women aged between 20 - 49 years, married or cohabiting with a male partner. Exclusion Critieria: school age childern(15 - 19 years), pregnant women, lactating mothers, virgins, menstruating women, wome with known gynaecological preoblems and menopasual women. | at least one schistosome ovum detected in cervical biospy tissue | Determine HIV - 1/2 Test and an immunochromatographic test | 3 | 19 | 31 | 278 | 1.416/ 0.0592 / 0.3965 to 5.0570 | None |  |
| Kjetland et al (2006) | Association between genital schistosomiasis and HIV in rural Zimbabwean women | October 2001 to June 2003, Zimbabwe | Cross sectional Design | Zimbabwe (North - west) | 445 | Female | Median age was 30 years for HIV-positive, and 36 years for HIV-negative women (P = 0.003). Women between the ages of 25 and 29 years had the highest HIV prevalence (45%) | All women aged 20–49 years were invited to take part in the study. Virgins, pregnant, postmenopausal and menstruating women were excluded. | Urine samples were examined for S. haematobium ova. A single terminal-spined ovum gave a positive diagnosis in Pap smears, wet mounts or biopsies of genital tissue | Serologic tests were run for HIV (29%, 153/523), | 29 | 96 | 41 | 279 | 2.0556 / 0.0076 / 1.2110 to 3.4893 | Age and BMI (logistic regression analysis and stratification by age groups done) |  |
| Ndhlovu et al, (2006) | Prevalence of urinary schistosomiasis and HIV in females living in a rural community of Zimbabwe: does age matter? | October 2001 to June 2003, Zimbabwe | Cross sectional Design | Mupfure and adjacent areas in Shamva District, Mashonaland Central Province, Zimbabwe | 544 | Female | The mean age was 33.2 years (range 15—49 years). | Women aged 15—49 years, non menopasual and sexually activie. | urine samples were collected on three consecutive days. Urine specimens were examined by the filtration technique | the Genelavia Mixt HIV-1/2 ELISA. The second test was the Recombigen HIV-1/2 enzyme immunoassay | 72 | 84 | 144 | 244 | 1.4524 / 0.0518 / 0.9970 to 2.1157 | Tribal origin, urban childhood and Age (Stratification by age groups done and multivariate analyasis controlling for age only done) |  |
| Kallestrup et al (2004) | Schistosomiasis and HIV-1 Infection in Rural Zimbabwe: Implications of Coinfection for Excretion of Eggs | October 2001 to June 2003 | Cross sectional Design | Mupfure and adjacent areas in Shamva District, Mashonaland Central Province, Zimbabwe | 1545 | Men and Women | Median (years) 37 years - HIV Negative and 34 - HIV Positive | Exclusion criteria were applied to participants presenting with clinical signs/symptoms of tuberculosis, terminal stages of schistosomiasis, or severe anemia and pregnant women | Microscopic examination of fixed volume urine samples filtered on Nytrel filters | rapid HIV-1/2 test kit (Determine) and confirmation was done with Oraquick or Capillus. | 110 | 297 | 316 | 822 | 0.9634 / 0.7740 / 0.7471 to 1,2425 | Egg counts, age, CD4 count, blood subsets. Adjustment to age, sex,CAA levels and schistisomes species by multiple regression done | Same Study but broken down in to the 2 different study designs used |
| Downs et al (2017) | Schistosomiasis and Human Immunodeficiency Virus in Men in Tanzania | Between April 2014 and February 2016. Tanzania | Cross sectional study | Nine rural villages in the Mwanza region of Tanzania | 674 | male | The median age was 34 years [interquartile range = 25–42]. | Men aged 18–50 years from nine different villages were invited to participate in this study. | We defined S. haematobium infection as any of the following: 1) S. haematobium ova in urine and/or 2) CAA 30 pg/mL in an S. haematobium endemic region in an individual with no S. mansoni ova in stool. | Rapid tests (SD Bioline) were used with confirmatory testing for positive samples (Unigold) as per the national testing algorithm. | 7 | 31 | 88 | 548 | 1.4062 / 0.4322 / 0.6007 to 3.2916 | Age, Years of school completed, number of sexual partners in the past 6 months, dyspareunia, number of people living in household, age in years after first sex, typical sex partners more than 5 years younger, ever treated for STI, hemospermia, painful genital ulcers, syphillis. multiple logistic regression to exmaine all these factors |  |
| Downs et al (2011) | Urogenital Schistosomiasis in Women of Reproductive Age in Tanzania’s Lake Victoria Region | conducted between August 2009 and May 2010, Tanzania | cross-sectional study | Lake Victoria in northwest Tanzania | 457 | Female | The median age was 30 years (interquartile range [IQR] = 24–35 years). | Women aged between 18 and 50 years . Women who were menstruating or refused gynecologic examination were excluded. | A single urine sample was collected from women between 10 AM and 2 PM, and it was filtered and examined microscopically for Schistosomal ova. Abnormal cervical lesions were biopsied. Specimens were stained with Hematoxylin and Eosin (H&E) for histopathological examination and Trypan Blue to examine for schistosomal ova using the crush technique | blood was collected and tested using a rapid test (SD Bioline, Standard Diagnostics,Inc., South Korea) | 4 | 23 | 19 | 411 | 3.762 / 0.0248 / 1.1827 to 11.9662 | Age, Gynaecological symptoms and all baseline characterisitcs (Marital status, number of children, people living in household, occupation, went to bed hungry, number of water contacts per day, ever treated for schistosomiasis, received artemesinin medication in past 3 years for malaria). Adjustment model catered for all the above during analysis. |  |
| Wall et al (2018) | Schistosomiasis is associated with incident HIV transmission and death in Zambia | 1994–2012 | (Cross sectional SD) | Lusakha, Zambia | 1046 | male | Age (mean, SD) - HIV + - Schist Ab positive : Negative 28.2 (7.3):28.3(6.9); HIV- Schist Ab Positive:Negative 27.6 (7.0): 26.6 (6.9) | Heterosexual HIV discordant couples (M+F- and M-F+) enrolled in an open cohort with longitudinal follow-up every three months in Lusaka, Zambia | A positive schistosomiasis result was defined as having a positive SWAP antibody response. Immunoblot testing using species-specific antigens was used to distinguish between S. mansoni and S. haematobium antibodies | rapid HIV antibody testing, | 381 | 218 | 309 | 138 | 0.7805 / 0.0625 / 0.6014 to 1.0130 | Age, sex, baseline schistosome specific antibody status of male partners, pregnant at baseline. Adjusment models were done to control for all the above factors. | Same study but broken down into men and female |
| Wall et al (2018) | Schistosomiasis is associated with incident HIV transmission and death in Zambia | 1994–2012 | (Cross sectional SD ) | Lusakha, Zambia | 1099 | female | Age (mean, SD) - HIV + - Schist Ab positive : Negative 28.2 (7.3):28.3(6.9); HIV- Schist Ab Positive:Negative 27.6 (7.0): 26.6 (6.9) | Heterosexual HIV discordant couples (M+F- and M-F+) enrolled in an open cohort with longitudinal follow-up every three months in Lusaka, Zambia | A positive schistosomiasis result was defined as having a positive SWAP antibody response. Immunoblot testing using species-specific antigens was used to distinguish between S. mansoni and S. haematobium antibodies | rapid HIV antibody testing, | 296 | 300 | 275 | 228 | 0.8180 / 0.0980 / 0.6448 to 1.0378 | Age, sex, baseline schistosome specific antibody status of male partners, pregnant at baseline. Adjusment models were done to control for all the above factors. | Same study but broken down into men and female |
| Kleppa et al (2015) | Schistosoma haematobium Infection and CD4+ T-Cell Levels: A Cross-Sectional Study of Young South African Women | May to October 2013. | Cross sectional SD | rural KwaZulu-Natal, South Africa. | 752 | Female | The mean age for HIV positive women was 20.1 (SD 2.4) years as opposed to 18.7 (SD 2.1) years for the HIV negative. | All women who were sexually active and above the age of 16 years were invited to participate in the study. Pregnant women were not investigated. | Schistosome eggs in urine were counted by microscopy | Bioline Rapid Test HIV and confirmatory Sensa Tri- Line HIV Test Kit. | 21 | 100 | 127 | 504 | 0.8334 / 0.4829 / 0.5009 to 1.3866 | CD4 count, sandy patches, abnormal blood vessels and schistosome eggs in urine. Adjustment during analsis was done for all the above. | Though Sample size is 765, only data for 752 participants was provided for Schist and HIV association data |
