# Supplementary material for: A systematic review and meta-analysis to assess the association between urogenital schistosomiasis and HIV/AIDS infection
Source: PLoS Negl Trop Dis. 2020 Jun 15;14(6):e0008383. doi: 10.1371/journal.pntd.0008383 (PMC7316344; doi:10.1371/journal.pntd.0008383)
Supplement: S8 Appendix — (DOCX) [file pntd.0008383.s008.docx]

| **Author (Year of publication)** | **Article title** | **Quality assessment - EPHPP QA Tool** |
| --- | --- | --- |
| Yirenya - Tawiah et al (2009) | HIV testing in community based research a case study of female genital schistosomiasis and HIV in the Volta Basin of Ghana | Selection Bias: Q1 - 1, Q2 - 5. Rate - **Moderate - 2**. Confounders: Q1 - 3, Q2 - 4. Rating: Weak (3). Blinding: Q1 - 3, Q2 -3. Rate: **Weak (3)**. Data Collection: Q1 - 1, Q2 - 2. Rate: **Moderate**. Withdrawal and Dropouts: Q1 - 3, Q2 - 5. Rate: **Moderate.** Intervention Integrity: Q1 - 4, Q2 - 2, Q3- 5. Rate: **Weak (3)**. Analyses: Q1 - Individual, Q2 - Individual, Q3 - 3, Q4 - 3. Rate: **Weak (3). Overall Grade: Weak** |
| Kallestrup., et al 2006 | Schistosomiasis and HIV-1 Infection in Rural Zimbabwe: Implications of Coinfection for Excretion of Eggs | A: Q1 - 1, Q2 - 2. Rate: **Strong**. C: Q1 - 1, Q2 - 1. Rate: **Strong.** D: Q1 - 2, Q2 - 1. Rate: **Weak**. E: Q1 - 1, Q2 - 2. Rate: **Strong.** F: Q1 - 5, Q2 - 5. Rate: **Not Applicable.** G: Q1- 3, Q2 - 1, Q3 - 5. Rate: **Moderate**. H: Q1 - Individual, Q2 - Individual, Q3 - 1, Q4 - 2. Rate: **Moderate.** **Overall Rate: Moderate** |
| Wall et al (2018) | Schistosomiasis is associated with incident HIV transmission and death in Zambia | A: Q1 - 1, Q2 - 1. Rate: **Strong**. C: Q1 - 1, Q2 - 1. Rate: **Strong.** D: Q1 - 2, Q2 - 1. Rate: **Weak**. E: Q1 - 1, Q2 - 2. Rate: **Strong.** F: Q1 - 1, Q2 - 1. Rate: **Strong.** G: Q1- 3, Q2 - 1, Q3 - 5. Rate: **Moderate**. H: Q1 - Individual, Q2 - Individual, Q3 - 1, Q4 - 3. Rate: **Moderate.** **Overall Rate: Moderate** |
| Downs et al (2011) | Urogenital Schistosomiasis in Women of Reproductive Age in Tanzania’s Lake Victoria Region | A: Q1 - 1, Q2 - 1. Rate: **Strong (2)**. C: Q1 - 1, Q2 - 1. Rate: **Strong**. D: 2, Q2 - 1. Rate: **Weak**. E: Q1 - 1, Q2 - 1. Rate: **Strong**. F: N/A. G: Q1 - 3, Q2 - 1, Q3 - 5. Rate: **Moderate**. H: Q1 - Individual, Q2 - Individual, Q3 - 1, Q4 - 2. Rate: **Moderate.** **Overall Grade: Moderate** |
| Downs et al (2017) | Schistosomiasis and Human Immunodeficiency Virus in Men in Tanzania | A: Q1 - 1, Q2 - 1. Rate: Rate: **Strong (1)**. C: Q1 - 2, Q2 - 2. Rate: **Moderate**. D:Q1 - 2, Q2 - 1. Rate: **Moderate**. E: Q1 - 1, Q2: 2. Rate: **Strong**. F: Q1 - 4, Q2 - 5. Rate: N/A. G: Q1 - 3, Q2 - 1, Q3 - 5. Rate: **Moderate** (2). H: Q1 -Individual, Q2 - Individual, Q3 - 6, Q4 - 1. Rate: **Moderate.** **Overall Grade: STRONG** |

| **Author (Year of publication)** | **Article title** | **Quality assessment - EPHPP QA Tool** |
| --- | --- | --- |
| Kleppa et al (2015) | Schistosoma haematobium Infection and CD4+ T-Cell Levels: A Cross-Sectional Study of Young South African Women | A: Q1 - 1, Q2 - 5, Rate: **Moderate**. C: Q1 - 3, Q2 - 4, Rate: **WEAK** (2). D: Q1 - 2, Q2 - 1. Rate: **Moderate (2**). E: Q1 - 1, Q2 - 1. Rate: **Strong** (1). F: Q1 - 4, Q2 - 5. Rate: **N/A**. G: Q1 - 3, Q2 - 1, Q3 - 5. Rate: **Moderate (2**). H: Q1 - Individual, Q2 - Individual, Q3 - 1, Q4 - 3. Rate: **Moderate (2)** **Overall Rate MODERATE** |
| Ndhlovu et al, (2006) | Prevalence of urinary schistosomiasis and HIV in females living in a rural community of Zimbabwe: does age matter? | A: Q1 - 1, Q2 - 1, Rate: **Strong (1)**. C: Q1 - 3, Q2 - 4. Rate: **Weak (3)**. D: Q1 - 1, Q2 - 1. Rating: **Strong (1)**. E: Q1- 1, Q2 - 1. Rating: **Strong (1)**. E: Q1 - 4, Q2-5. N/A. G: Q1- 3, Q2 - 1, Q3 - 5. Rating: **Moderate (2)**. H: Q1 - Individual, Q2 - Individual, Q3 - 1, Q4 - 3. Rating: **Moderate (2). Overall Rating: Moderate** |
| Kjetland et al (2006) | Association between genital schistosomiasis and HIV in rural Zimbabwean women | Selection Bias: Q1 - 1, Q2 - 1. Rating: **Strong (1).** Confounders: Q1 - 1, Q2 - 2. Rate: **Moderate (2).** Blinding: Q1 - 3, Q2 - 1. Rate: **Moderate (2).** Data Collection: Q1 -1, Q2 - 2. Rate: **Strong (1).** Withdrawals and Dropouts: Q1 - 2, Q2 - 4. Rate: **Moderate (2).** Intervention Integrity: Q1 - 3, Q2 - 2, Q3 - 5. Rate: **Moderate (2).** Analyses: Q1 - Individual, Q2 - individual, Q3 - 1, Q4 - 3. Rate: **Moderate (2).** **Overall Rating: Strong** |
